# Reply: TIMP-1 enhancer sequence – real or bacterial?

**DOI:** 10.1038/sj.bjc.6601354

**Published:** 2003-10-28

**Authors:** M E Stearns, Y Hu, M Wang

**Affiliations:** 1 Department of Pathology and Laboratory Medicine, Drexel University College of Medicine, Mail Stop 435, 15th and Vine Sts, Philadelphia, PA 19102-1192, USA

**Sir**,

The characterisation of the TIMP-1 gene was published in Wang *et al* (1998). The TIMP-1 gene was cloned from PC-3 ML genomic DNA. The *Pst*1–*Xba*1 fragment at the 5′end of the Pst1 4.0-kilobase TIMP-1 genomic clone was subcloned into plasmid PUC 19. This fragment was sequenced from both ends on opposite strands with the Sequenase Virgin 2.0 sequencing system (US Biochemical). Each strand was sequenced with both the Klenow fragment and reverse transcriptase by standard dideoxy procedures. Blast analysis of the 5′ promoter region revealed a 97% homology with *Homo sapiens* TIMP-1 promoter region (chromosome X genomic contig, NT 011568). Blast analysis of the coding region revealed a 97% homology with human TIMP-1 (Docherty *et al*, 1985) and 96% homology with the coding region of the *E. coli* TIMP-1.
